# Repeated-dose toxicity of common ragweed on rats

**DOI:** 10.1371/journal.pone.0176818

**Published:** 2017-05-04

**Authors:** Tivadar Kiss, Andrea Szabó, Gábor Oszlánczi, Anita Lukács, Zoltán Tímár, László Tiszlavicz, Dezső Csupor

**Affiliations:** 1 University of Szeged, Faculty of Pharmacy, Department of Pharmacognosy, Szeged, Hungary; 2 University of Szeged, Interdisciplinary Centre for Natural Products, Szeged, Hungary; 3 University of Szeged, Faculty of Medicine, Department of Public Health, Szeged, Hungary; 4 SOLVO Biotechnology, Szeged, Hungary; 5 University of Szeged, Faculty of Medicine, Department of Pathology, Szeged, Hungary; Hungarian Academy of Sciences, HUNGARY

## Abstract

*Ambrosia artemisiifolia* L. is an invasive species with highly allergenic pollens. Ragweed originates from North America, but it also occurs and is spreading in Europe, causing seasonal allergic rhinitis for millions of people. Recently, the herb of *A*. *artemisiifolia* has gained popularity as medicinal plant and food. The effects of its long-term intake are unknown; there are no toxicological data to support the safe use of this plant. The aim of our study was to assess the repeated dose toxicity of *A*. *artemisiifolia* on animals. Ragweed puree was administered in low dose (500 mg/kg b. w.) and high dose (1000 mg/kg b. w.) to male Wistar rats according to 407 OECD Guidelines for the Testing of Chemicals. Clinical symptoms, various blood chemical parameters, body weight and organ weights of the rats were measured. Reduced liver function enzymes (AST, ALT), reduced triglyceride level in the low dose and increased carbamide level in the high dose group were observed. The weight of the liver relative to body weight was significantly reduced in both groups, while the brain weight relative to body weight was significantly elevated in both groups. According to our results, the repeated use of ragweed resulted in toxic effects in rats and these results question the safety of long-term human consumption of common ragweed.

## Introduction

Common ragweed *(Ambrosia artemisiifolia* L., Asteraceae) is an annual plant with high allergenic potential. At present, 33 million people are sensitized to ragweed in Europe [1] and 23 million in the US [2]. Frederick W. Heyl reported in 1917 that it caused hay fever [3], however the reason for this immune reaction was revealed only half a century later. The major allergens of the pollen are peptides with immunoglobulin-E binding capacity *(Amb a 1* and *Amb a 2* endopeptidases); they trigger rhinitis, oculorhinitis and other symptoms of hay fever [4,5]. The mechanism of the immune reaction is influenced by the lipid content of the pollen [6]. Dermal exposure to the plant can cause contact dermatitis, which has previously been described also for other plants belonging to Asteraceae. This reaction is due to the sesquiterpene lactones, characteristic marker compounds of this family [7,8].

The phylogenesis of the *Ambrosia* genus took place at the Sonoran Desert (USA). Later the genus radiated outwards to the territories of North America and Mexico which is now considered as a native region of the *A*. *artemisiifolia* [9,10]. The first seeds arrived in Europe around 1860 probably with clover seed grains [11]. In the 1930’s it was introduced to China [12,13]. Nowadays it is widespread in Europe (Hungary, former Yugoslavian countries, France, Switzerland, Germany and Russia), Japan, South Korea, Australia, New Zealand, Central and South America [14]. The fast spreading of *A*. *artemisiifolia* can be explained by its wide ecological niche, which meets the environmental conditions of the aforementioned territories. This fact makes common ragweed one of the most invasive species in the world [15,16]. There is some evidence that the infection of new territories might speed up as a consequence of global warming [17–20] and the great genetic variability of ragweed.

Although some ethnobotanical sources reported that *A*. *artemisiifolia* was used by Native Americans for medicinal purposes, however these publications were focusing mainly on ethnographic aspects without discussing pharmacological background [21–28]. The widespread and long-standing folk medicinal application of this plant is not supported by available data. However, common ragweed had never been part of the folk medicine in Europe, yet the medicinal use of the herb (usually collected before the flowering period) has started recently and is spreading fast. To the best of our knowledge, neither effects of long term consumption, nor the expression of allergens *(Amb a 1* and *2)* has been examined, thus the risks of applying the herb for medicinal purposes is unknown.

**Fig 1:**
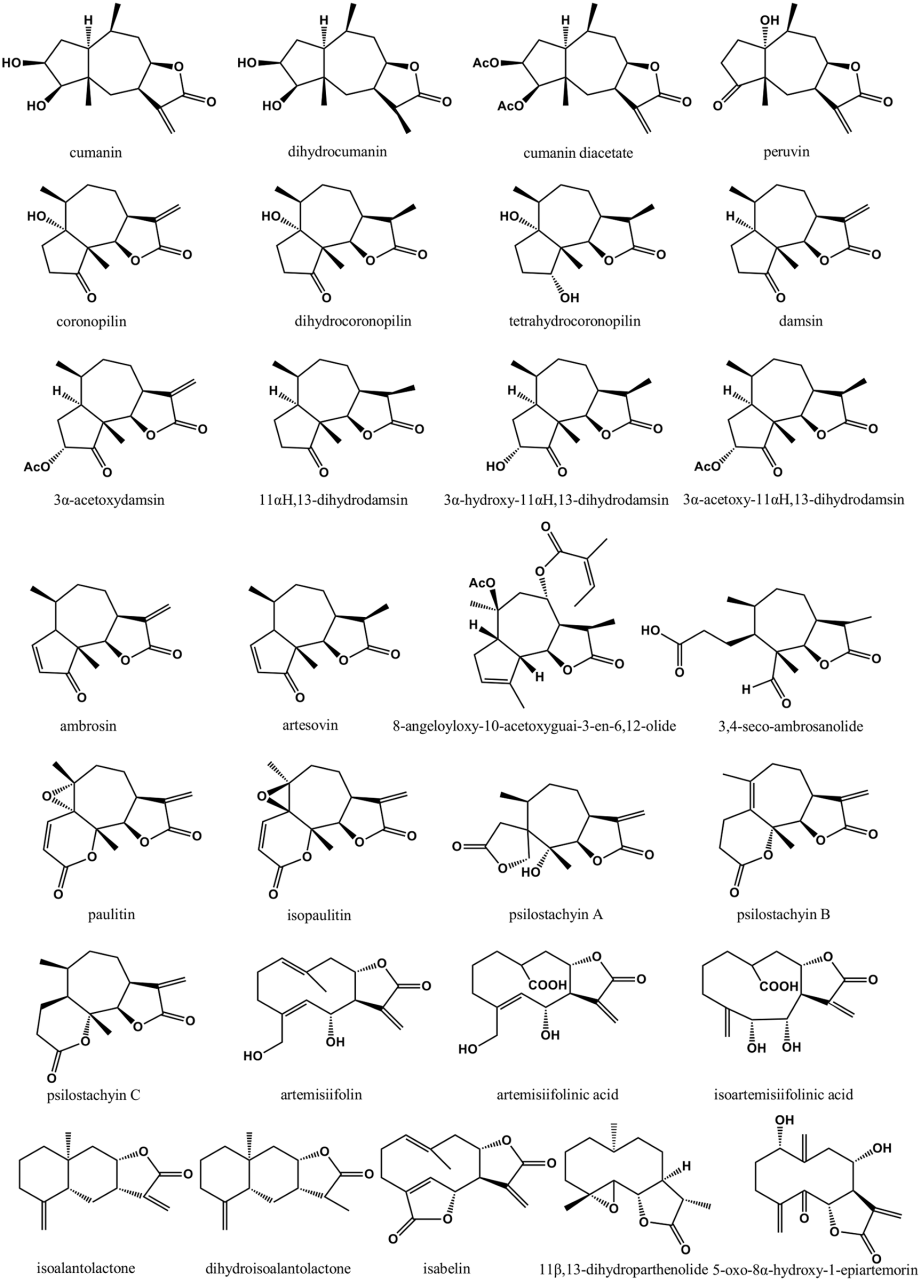
Sesquiterpene lactones isolated from *A*. *artemisiifolia*.

Preclinical investigations of *A*. *artemisiifolia* were mainly conducted with its isolated compounds. According to the literature, 29 sesquiterpene lactones have been isolated from *A*. *artemisiifolia* (Fig 1) [29–41] and many of them were reported to have noteworthy pharmacological activities (Table 1), such as antibacterial, antifungal, antiprotozoal, anti-inflammatory, cardiovascular and hepatoprotective effects.

**Table 1:** Bioactivity of ambrosian sesquiterpene lactones.

Compound	Effects				
	antileukemic and anti-lymphoma	anti-cancer	antiparasitic, insecticid	antimicrobial	other
ambrosin	Jurkat cell line [42] P-388 [43]	NF-κB inhibitor[44]	molluscicidal [45]		antiarrhythmic effect [46] allergenic [47]
coronopilin	Jurkat cell line [48] U937 [48]	NF-κB inhibitor [49] SAT3 activator [49]	larvicidal [50]		allergenic [51]
cumanin	BW5147 [52]		trypanocidal [53] antileishmanial [53]		anti-inflammatory [54]
damsin		NF-κB inhibitor [44,49] SAT3 activator [49] prostate cancer (DU145) [55]	antileishmanial [55] trypanocidal [55]	antituberculotic [55] antifungal [56]	antyarrhytmic [46] allergenic [51]
dihydrocoronopilin				antibacterial [57]	
dihydroisoalantolactone			larvicidal [58]		
isoalantolactone	K562/A02 [59,60] deregulates Myb [61]	human breast cancer cells (MCF-7 [62–64], KT [62,63,65], MDA-MB-231 [64]) hepatocellular carcinoma [66] (HLE) [65,67] gynecological cancer (HEC-1 [68], HOC-21 [68], HAC-2 [68], HeLA [69,70], SKOV3 [71]) osteosarcoma (U2OS [72]) NF-κB [72] Nrf2/ARE activator [73,74] pancreatic carcinoma-1 (PANC-I) [75] human gastric adenoma (MK-1 [69], SGC-7901 [76]) melanoma [69] human colorectal cells (HCT116) [77] glial cell line U251SP [65], T-98 [65] head and neck squamous cell carcinoma (HNSCC) [78] non-small-cell lung carcinoma [66]	larvicidal [50]	antibacterial [79,80] antituberculotic [81]	allergenic [34]
isopaulitin		human breast cancer cells (MCF-7 [82], BCI [82]) epidermoid carcinoma (A-431 [82], KB [82]) human colon cancer (Lu1 [82]) human lung cancer (Col) [82]			
paulitin		human breast cancer cells (MCF-7 [82], BCI [82]) epidermoid carcinoma (A-431 [82], KB [82]) human colon cancer (Lu1 [82]) human lung cancer (Col) [82]			
peruvin	BW5147 [52]	breast cancer (aromatase inhibitor) [83]	trypanocidal [84,85] antileishmanial [84,85] antiplasmodial [86]		
psilostachyin A	BW5147 [52]		trypanocidal [84,85,87,88] antileishmania [84,85] antiplasmodial [86]		
psilostachyin B			trypanocidal [89]		
psilostachyin C	BW5147 [52]		trypanocidal [87,90]		

The antiproliferative effect of sesquiterpene lactones of the *Ambrosia* genus has been widely examined on various tumour cell lines.

The infection of novel territories by ragweed seems to be inevitable, hence public health and economic consequences will follow, which will be substantial. Beside its well-known allergic potential, the increasingly widespread use of common ragweed as food and medicinal plant [91–93] raises the safety concern. Several products are available on the market, typically as food supplements (dry ragweed powder [94], alcoholic extract [95]) or as food (puree made of the fresh buds of the plant [96]). The presumed (and advertised) beneficial effects of ingested ragweed include anxiolytic activity, strengthening of the immune system, detoxifying of the body, improving erectile functions, stimulating appetite, anticarcinogenic, anti-allergic, mucolytic effects [92,93]. There are no human studies to support these therapeutic indications. Considering the fact that *A*. *artemisiifolia* contains potentially cytotoxic sesquiterpene lactones, acute and chronic toxicological studies are necessary to establish its safety for human use. The aim of our study was to assess the repeated dose toxicity of a product containing pure of ragweed herb, using a rat model.

## Materials and methods

The analysed product (Keserű parlagfű készítmény 220 g—Tátra Sóbarlang Webáruház; in English: Bitter ragweed puree 220 g) was purchased online [97] in 2015. According to the product description, it contains a puree prepared from young and fresh ragweed herb and olive oil (the quantities of the components are not published). Solvents and additives were obtained from commercial sources (VWR, Sigma). All sections of this report adhere to the ARRIVE Guidelines for reporting animal research [98]. A completed ARRIVE guidelines checklist is included in supporting information (S1 File).

### Examination of the sesquiterpene lactone content

The presence of ragweed in the product was confirmed by the detection of sesquiterpene lactones characteristic to ragweed. 150 mg of the ragweed puree was extracted with 2 mL of *n*-hexane by ultrasonication for 10 minutes. The fraction rich in sesquiterpene lactones was extracted by solid phase extraction. A normal phase column (Thermo Scientific, HYPERSEP SI, 200 mg/3 mL, 60108–410) was conditioned with 9 mL of *n-*hexane. The hexane extract was loaded with 3 mL of *n-*hexane and washed with 6 mL *n*-hexane. Elution was then carried out with *n-*hexane (3 mL), ethyl acetate (15 mL, fractions), dichloromethane (9 mL) and methanol (9 mL).

These fractions were screened for sesquiterpene lactone content by thin layer chromatography. Thin layer chromatography was carried out at room temperature on silica gel (SiO_2_ 60 F_254_, Merck 1.05554.0001) and toluene—ethyl acetate—formic acid 5:4:1 was applied as mobile phase. Dried plates were sprayed with *cc*.H_2_SO_4_ and heated in an oven set at 110°C for 5 minutes [99].

The presence of sesquiterpene lactones was confirmed by LC-MS. The dry residue of the analysed fraction was dissolved in methanol-water 2:1 and filtered on 0.45 μm nylon filter. The volume of the injected sample was 5 μL. Chromatographic separation was performed with an Agilent 1260 HPLC equipped with a reversed phase Agilent 3.0 x 50 mm 2.7 μm column (Agilent Poroshell 120 EC-C18). Mobile phase consisted of 0.1% formic acid in LC-MS quality water (eluent A) and 0.1% formic acid in LC-MS quality acetonitrile (eluent B). Gradient elution was applied (eluent B 5-40-95-5-5% in 0-3-7-20-20.1–22 minutes). The flow rate was 0.5 mL/min. The column temperature was set up to 40°C. The HPLC was coupled to an Agilent 6460A Triple Quadrupole Mass Spectrometer. MS detection was carried out in full scan mode (*m/z* range: 50–2000, fragmentor: 75 V, positive polarity, standard JetStream ion source settings). Data acquired and evaluated using MassHunter software v. B.03. Expected *m/z* values were extracted from the total ion current chromatogram.

### Animals and husbandry

Altogether 24 male SPF Wistar rats were included in the experiments (purchased from Toxi-Coop Zrt., Hungary), weighed 170–200 g (40–43 days old) at the start of the study. After one week of acclimatization the rats were randomized according to body weight into three groups (control [CON], low dose [LD], high dose [HD], 8 animals/group). For statistical and ethical reasons each group consisted of 8 animals, providing enough data for statistical analysis but minimizing the number of animals used. As environmental enrichment we used unbleached, clean paper tubes. Dust free wood shavings were applied as bedding material. Rats were kept under standard climatic conditions (22–24°C, 12 h light/dark cycle with light starting at 6:00 a.m., 2–3 rats in one cage) with free access to drinking water. Rats were fed with standard, certified rodent chow. During the whole procedure, the regulations of the Hungarian Act No. XXVIII of year 1998 on protection and care of animals were strictly followed.

The protocols used in this experiment for handling, treatment or anaesthesia was approved by the Committee on the Ethics of Animal Experiments of the University of Szeged and the Directorate of Food Safety and Animal Health Care, Government Agency of Csongrád County (Permit number: XXI./151/2013.) All efforts were made to minimize suffering.

### Ragweed administration

During this study the 407 OECD Guidelines for the Testing of Chemicals, repeated dose 28-day oral toxicity study in rodents, was followed [100].

During the 28-day study, the body weights of animals were measured every morning. According to the measured body weight (S2 File) individual special feed portions (self-made sugar cookie balls as vehicle) with or without ragweed puree were prepared and then given to each animal individually while they are left in their home cage (S3 File).

The basic recipe for sugar cookie dough included 55% plain flour, 20% caster sugar and 25% water. Control animals received plain cookie dough without ragweed (0 m/m% in dough). LD animals received 500 mg/kg b. w. ragweed (12.5 m/m% in dough, replacing half of the water), while HD rats were given 1000 mg/kg b. w. ragweed (25 m/m% in dough, replacing the whole amount of water). All animals received 4 g cookie dough/kg b. w. The doughs were prepared once a week and kept refrigerated thereafter. As no previous animal study was found in the literature with ragweed puree doses, they were determined according to the following toxicological calculations. Dosage recommendation on the label of the ragweed puree (daily 1 teaspoon taken in the morning on an empty stomach) was considered as human median effective dose (ED_50_). According to product description one bottle of ragweed puree (220 g) is enough for one month (30 days), consequently 7.3 g per day. Considering this amount to an average adult (approximately 70 kg b. w.) the human ED_50_ for this ragweed puree is 100 mg/kg b. w. As this is the first toxicological study with ragweed puree, we corresponded human ED_50_ to animal HD by a calculation with a multiplier of 10 (safety factor accounting human-animal extrapolation) leading to 1000 mg/kg b. w. According to 407 OECD Guideline at least two-fold interval should be between dosages, therefore the LD was determined as 500 mg/kg b. w.

During the acclimatization period all rats habituated to the cookie balls (preventing neophobia) and were trained to accept voluntarily the cookie balls from gloved fingers of the researcher, who always made sure that the whole ball was eaten. The access to standard rodent chow was restricted for 2 hours per day after treatment. On one hand this was a reward, a positive confirmation for the animals after the successful treatment; and on the other hand they became very hungry by the next morning, that further motivated them to accept the cookie balls. Oral administration was used instead of traditional gavage technique in order to cause the least stress and harm to the animals [101,102], and to model human exposure the most objectively. The feeding performance was 100% during the treatment period; no uneaten cookie dough was noticed.

### Measurements

As the experimental outcome of the study clinical symptoms, body weight changes, organ weights changes and blood chemistry was mastered. General clinical observations were done every day looking for abnormal signs, symptoms, morbidity or mortality. Detailed clinical observation was done once before the first exposure and once a week thereafter. These observations were made outside the home cage usually after the rodent chow feeding period at each occasion. Every signs and symptoms were recorded including appearance (nutritional status, fur, eyes, ears, whiskery pad, nostrils, legs and tail), behaviour (activity, posture, temperament, faeces and urine), respiration (secretion, crepitation), circulation (temperature and colour of hind limbs, whiskery pad or tail) and nervous system effects (reflexes, reactions).

After the treatment period the animals were over-anaesthetized with isoflurane inhalation. Using a precision vaporizer with induction chamber and waste gas scavenger, the isoflurane gas was administered slowly up to >5% in 100% oxygen and continued until lack of respiration for >1 minute was observed. The rats were dissected and blood samples were taken immediately from vena cava. Then, the main organs were removed and weighed (brain, liver, lungs, heart, kidneys, spleen, thymus and adrenals), as organ weight is a sensitive basic toxicological indicator. Since absolute organ weight is influenced by the whole body weight, therefore organ-to-body weight ratio (related to 100 g b.w.) and—in case of neurotoxic substances potentially affecting brain weight—organ-to-brain weight ratio was calculated [103].

From blood samples, serum was separated for estimation of the various blood chemical parameters, levels of cholesterol, triglyceride, high-density lipoprotein (HDL), low-density lipoprotein (LDL), alanine aminotransferase (ALT), aspartate aminotransferase (AST), alkaline phosphatase (ALP), gamma-glutamyl transferase (GGT), bilirubin, carbamide, creatinine; number of leukocytes, number and proportion of neutrophils, lymphocytes, monocytes, eosinophils, basophils; number of erythrocytes, haemoglobin, haematocrit, mean corpuscular volume (MCV), mean corpuscular haemoglobin MCH, mean corpuscular haemoglobin concentration (MCHC), red cell distribution width (RDW-CV), number of thrombocytes, and mean platelet volume (MPV).

### Statistical analysis

The distribution of data was checked for normality by Kolmogorov-Smirnov test. In case of normal distribution one-way ANOVA and post hoc LSD test (p<0.05) were used. When a variable was not normally distributed, Kruskal-Wallis test was used for evaluation. In case of significance (p<0.05) the data were tested using the Mann-Whitney test, to show which groups are significantly different from each other. The unit of analysis was individual data of individual animals. SPSS 23.0 software pack was used to the statistical analysis.

## Results

### Sesquiterpene lactone content of the product

The fractions obtained by solid phase extraction were examined by thin layer chromatography. The sesquiterpene lactones gave specific colour reaction [99]. The richest sesquiterpene lactone content was observed in the ethyl acetate fraction. The presence of sesquiterpene lactones, characteristic to ragweed (psilostachyin A, psilostachyin B, psilostachyin C, paulitin, isopaulitin, damsin, dihydrodamsin, hidroxydihydrodamsin, 3-acetoxydamsin) [34,38,104,105], was confirmed by LC-MS, reassuring the ragweed content of the analysed product.

### Clinical observation

There was no remarkable clinical symptom recorded during the observations. The only symptom that appeared in 1–4 animals/group/observational occasion is the lengthened latency time of balance reaction (the rat by head upside down is put onto the lowest part of a grid surface that is inclined in 30° and the animal has to move upward immediately). However, no statistical significance was seen among groups or between treatment weeks (S4 File).

### Blood chemistry

Among the biochemical parameters, the activity of liver function enzymes (AST, ALT), the level of triglyceride, carbamide and creatinine were altered significantly in the blood of treated rats. All other laboratory results showed no significant differences (S5 File).

**Fig 2:**
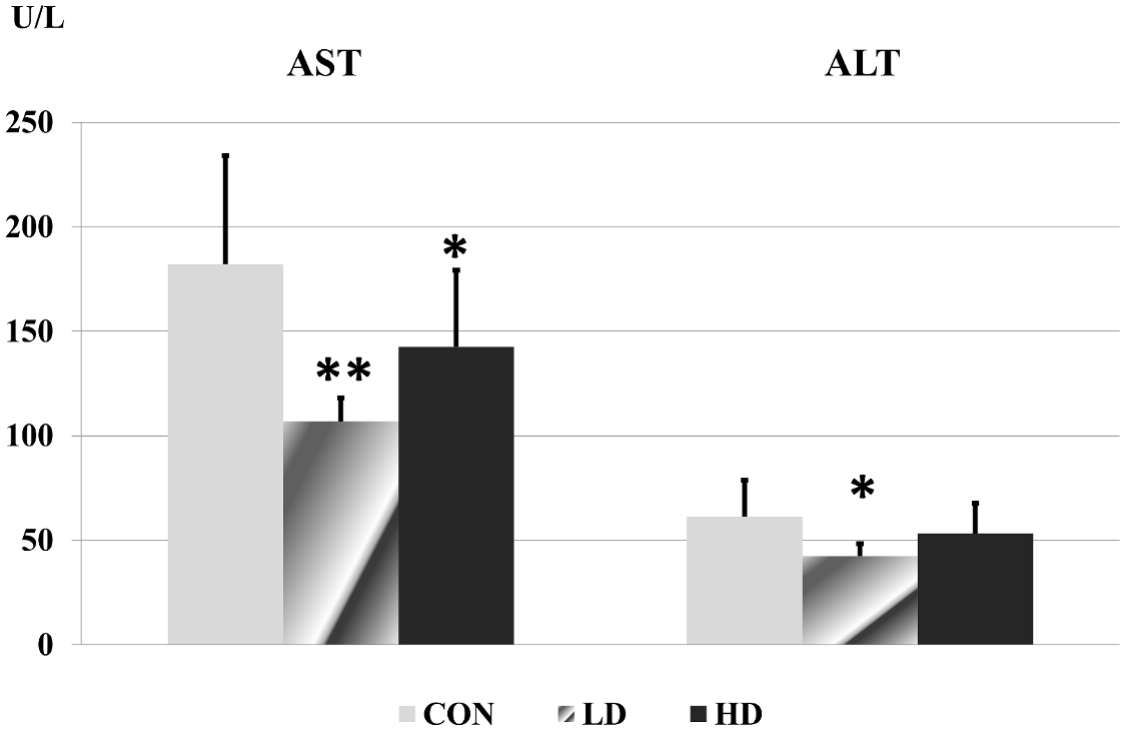
Levels of AST and ALT in blood serum samples of the control and treated rats. (CON: control; LD: low dose of ragweed; HD: high dose of ragweed; Mean ± S.D., n = 8; *: p<0.05 vs. control; **: p<0.01 vs. control.).

**Fig 3:**
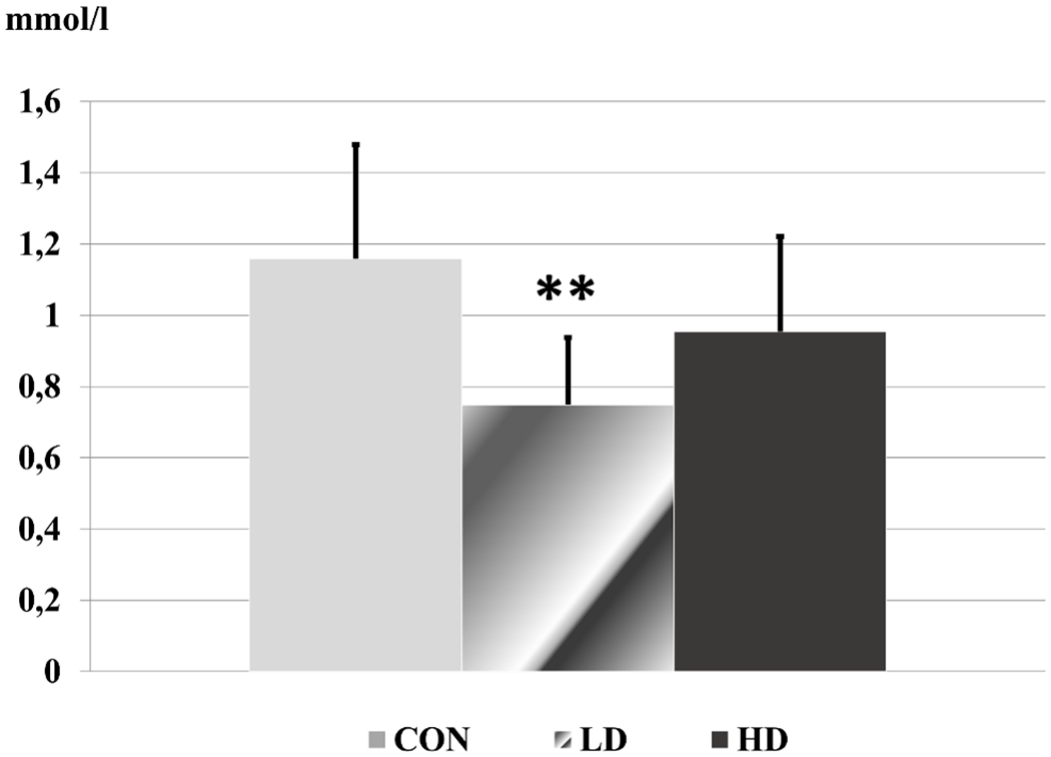
Triglyceride level in blood serum samples of the control and treated rats. (CON: control; LD: low dose of ragweed; HD: high dose of ragweed; Mean ± S.D., n = 8; **: p<0.01 vs. control.).

**Fig 4:**
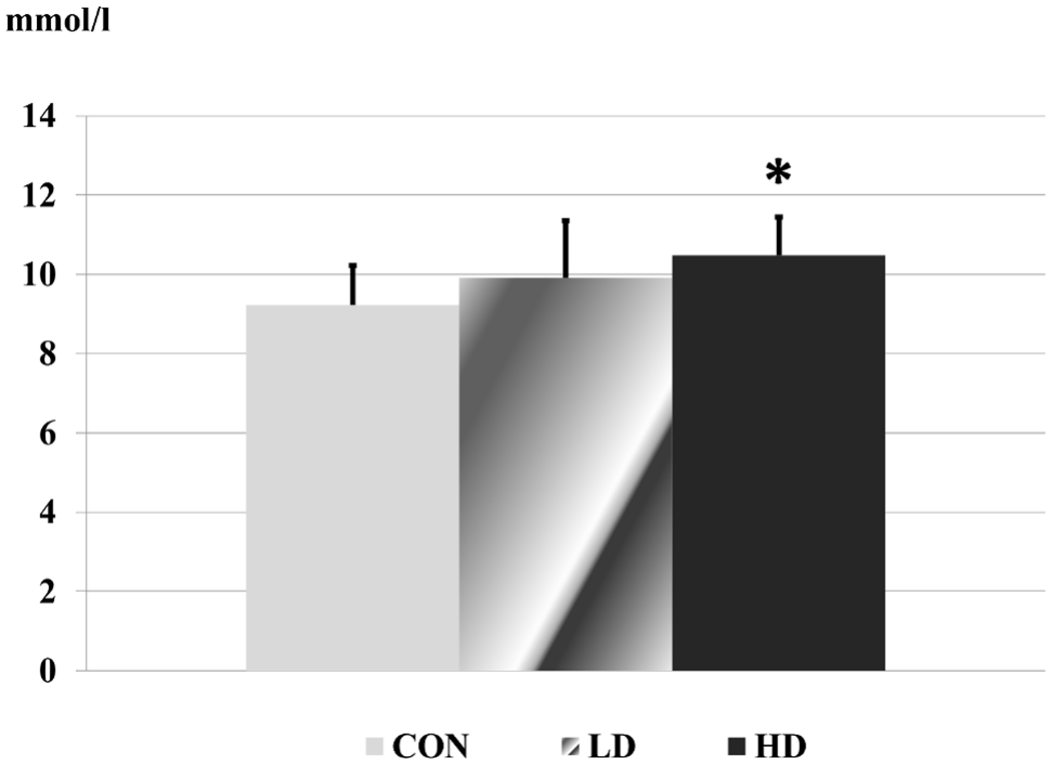
Carbamide level in blood serum samples of the control and treated rats. (CON: control; LD: low dose of ragweed; HD: high dose of ragweed; Mean ± S.D., n = 8 (6 in HD); *: p<0.05 vs. control.).

**Fig 5:**
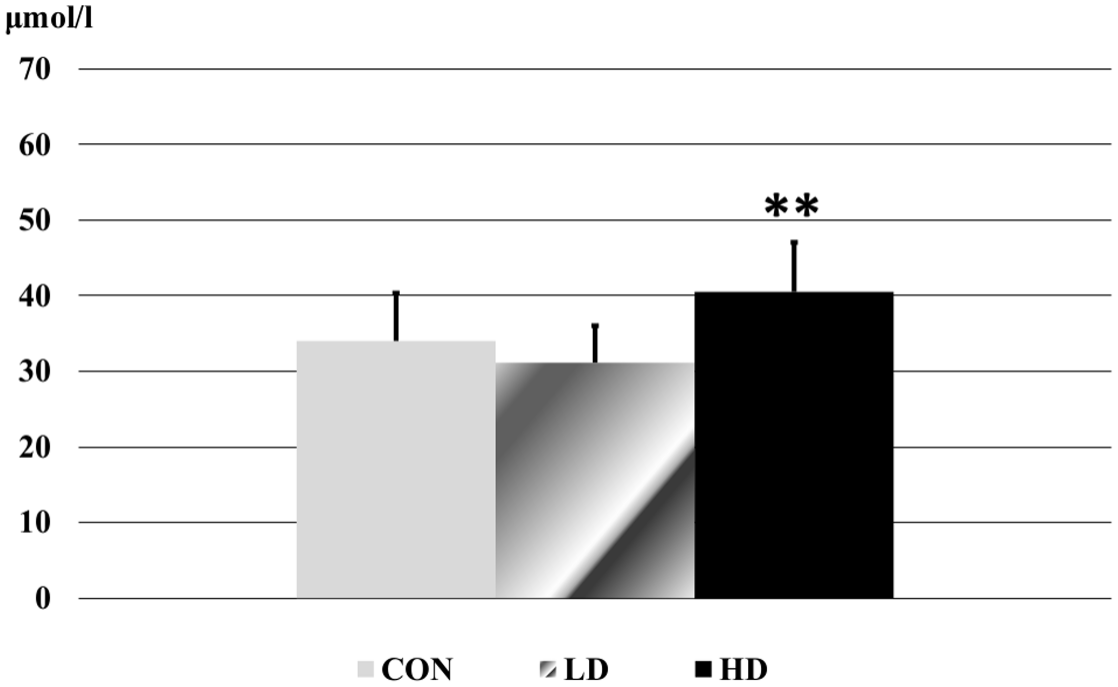
Creatinine level in blood serum samples of the control and treated rats. (CON: control; LD: low dose of ragweed; HD: high dose of ragweed; Mean ± S.D., n = 8 (6 in HD); **: p<0.01 vs. low dose.).

The activity of liver function enzymes reduced significantly in the low dose treated animals. In the high dose group the reduction was only significant in case of AST (Fig 2). The level of triglyceride in the blood decreased in the treated rats, which was significant in the low dose group (Fig 3). The carbamide level showed a dose-related elevation in the treated animals, the change became significant in the high dose ragweed group (Fig 4). In case of creatinine level significant difference occurred only between the two ragweed-treated groups (Fig 5).

### General toxicological parameters

Some weight gain difference was noticed in the treated rats compared to control, but there were no significant differences between the groups over time (without figure). The relative organ weights to 100 g body weight (Table 2) were calculated (S6 File). The weight of the liver declined with the dose and the difference was statistically significant. The relative weight of brain was significantly increased in both treatment groups compared to the control group.

**Table 2:** Relative organ weights to 100 g body weight in the control and treated animals.

Group	Relative organ weight to body weight							
	Heart	Thymus	Lung	Liver	Spleen	Kidney	Adrenal gland	Brain
Control	0.2479±0.0081	0.1077±0.0231	0.3472±0.0189	3.9091±0.1221	0.2149±0.0300	0.6013±0.0629	0.0157±0.0018	0.5381±0.0206
Low dose of ragweed (500 mg/kg)	0.2549±0.0143	0.1120±0.0245	0.3587±0.0339	3.5440*±0.1192	0.2403±0.0447	0.6289±0.0502	0.0169±0.0022	0.5950*±0.0464
High dose of ragweed (1000 mg/kg)	0.2592±0.0177	0.0977±0.0217	0.3665±0.0301	3.4527**±0.3694	0.2339±0.0222	0.6211±0.	0.0184±0.0027	0.5928*±0.0289

Mean ± S.D., n = 8 (6 in HD).

* p<0.05 vs. control,

** p<0.01 vs. control.

Relative organ weight to body weight: organ weight divided by the body weight multiplied by 100.

As the brain weight changed by treatment, hence the relative organ weights to brain weight were calculated (Table 3). The relative weight of the liver remained significantly decreased in the treatment groups versus control. The effect on other organ weights was negligible.

**Table 3:** Relative organ weights to brain weight in the control and treated animals.

Group	Relative organ weights to brain weight						
	Heart	Thymus	Lung	Liver	Spleen	Kidney	Adrenal gland
Control	0.4614±0.0265	0.1996±0.0397	0.6459±0.0418	7.2761±0.4110	0.3988±0.0490	1.1186±0.1202	0.0293±0.0037
Low dose of ragweed (500 mg/kg)	0.4294±0.0204	0.1886±0.0418	0.6038±0.0462	5.9952**±0.5899	0.4044±0.0696	1.0599±0.0870	0.0284±0.0022
High dose of ragweed (1000 mg/kg)	0.4377±0.0300	0.1677±0.0333	0.6186±0.0462	5.8620**±0.9099	0.3956±0.0440	1.0628±0.0683	0.0311±0.0046

Mean ± S.D., n = 8 (6 in HD).

** p<0.01 vs. control.

Relative organ weight to brain weight: organ weight divided by the brain weight.

By dissection and macroscopic observation of the organs it was found that two animals in the high dose group (animal identification number 20 and 22) had large, pale, smooth, hydronephrotic kidneys with expanded medulla and cortex containing numerous 2–15 mm diameter cysts. For further analysis these kidneys were sent to a histopathological examination where polycystic kidney disease (PKD) was confirmed (S7 File). As PKD is caused by genetic mutations both in humans and rats [106], therefore the presence of this disease was excluded from the evaluation of the effects of ragweed puree. After this finding the statistical analysis of the parameters affected by the kidneys was re-evaluated by excluding the values of the two affected animals, though the carbamide and creatinine level remained significantly elevated in the high dose group.

## Discussion

The study design and the special feeding technique were proven successful and were able to model the human oral ragweed puree exposure. Four weeks of treatment evoked no visible clinical symptom but was enough to induce some laboratory alterations that raise concern.

AST and ALT are considered two of the most important enzymes that indicate liver injury. Generally increased AST and ALT levels are associated with liver cell damage [107]; during the destruction of liver cells a peak in AST and ALT elevation occurs, but as the process progresses, enzyme levels may decrease even to the normal level [108]. In our study decreased enzyme levels were observed in the LD group, which may implicate a developing liver atrophy. This correlates with the observed reduction of relative liver weight. As no histological studies were made further experiments are required, although no macroscopic alteration could be observed on the liver tissue.

However, the treatment may also have hepatoprotective effects, since the hepatoprotective properties (normalization of ALT and AlkP activity, blood bilirubin level) of polyphenolic fractions isolated from *Ambrosia* were previously observed in the model of acute toxic hepatic damage caused by carbon tetrachloride in rats [109].

Parkhomenko *et al*. observed hypolipaemic properties of polyphenolic fractions isolated from common ragweed in rats after inducing hyperlipidaemia by the joint per oral administration of vitamin D_2_ and cholesterol [109]. Their results are in agreement with the documented triglyceride level reduction in our study.

Nephrotoxicity can be associated with a temporary elevation of laboratory values like carbamide, reflecting less carbamide being filtered through the kidneys. In our study the elevated carbamide levels were measured after ragweed treatment, which is a clear sign of kidney damage. In the study of Noori *et al*. Wistar rats were injected with an ethanolic extract of *Artemisia deserti* Krasch. (species related to *A*. *artemisiifolia)*. The extract produced histopathological alterations in the kidney of rats. The serum carbamide level was elevated, but the change was not significant [110]. Similarly to our study, levels of urea were increased significantly and kidney tissue damage was observed in the treated groups compared to control group in the experiment of Ene-ojo *et al*. [111]. However, we also evaluated the changes in creatinine levels and a similar trend was observed, but the change was significant only between the LD and HD groups.

Sesquiterpene lactones, found mainly in the Asteraceae family, may play a significant role in the toxic effects of ragweed on animal organs. Nephrotoxic effects of sesquiterpene lactone-containing herbal extracts have been reported in previous studies. The most widely known Asteraceae plant for its nephrotoxic effect is *Hymenoxys odorata* DC. Ingestion of this plant results in complex toxic symptoms, including glomerulonephrosis and hepatotoxicity in sheep and goats [112]. Parthenin, a compound characteristic to *Parthenium* species, inhibited RNA, DNA and protein synthesis *in vitro* in cultured bovine kidney cells [113]. In a repeated dose toxicity study on rats, the administration of sesquiterpene lactone containing extracts of *Smallanthus sonchifolius* (Poepp. & Endl.) H. Robinson resulted in alterations of creatinine, glucose and albumin levels, implying renal damage. Histological analysis showed lesions compatible with chronic glomerulonephrosis [114]. Similar nephrotoxicity, together with the signs of hepatotoxicity was observed in an acute toxicity study carried out on rats with the ethanolic extract of *Tithonia diversifolia* (Hemsl.) A. Gray, a species belonging to Asteraceae with confirmed sesquiterpene lactone content [115]. However, some sesquiterpene lactones may have hepatoprotective effects: an enriched fraction of sesquiterpene lactones of *Taraxacum officinale* L. roots exerted protective effect against carbon tetrachloride-induced hepatotoxicity in mice [116]. Certain sesquiterpene lactones of *Sarcandra glabra* (Thunb.) Nakai showed hepatoprotective activity against d-galactosamine-induced toxicity in WB-F344 rat hepatic epithelial cells *in vitro* [117].

Observations on livestock lead to the recognition that some sesquiterpene lactones are responsible for pharmacological effect on the central nervous system. Symptoms similar to Parkinson disease occurred in horses after long term feeding of *Centaurea solstitialis* L. and *C*. *repens* L. This disease—nigropallidal encephalomalacia—is characterized by liquefactive necrosis on various parts of the brain.

An other group of sesquiterpene lactones exerts neurotoxic activity by acting on GABA and glycine receptors as antagonists [112]. However, there are no data on the effect of sesquiterpene lactones on the brain weight. As organ weight change often precedes morphological alterations [118], our results draw attention to this possible effect, however, there is no evidence that the observed change in brain weight is linked to the sesquiterpenes of ragweed.

Our results refer to the toxic effect of common ragweed on kidney and its controversial effect on brain tissue. These results are in line with previous studies carried out with some other species of the genus *Artemisia* and with the Asteraceae family. The mechanism by which the ragweed constituents affect different organs cannot be derived from the present study, the reason for these toxic effects remains to be clarified.

## Conclusion

In this experiment some protective effects on the liver and on triglyceride level were observed during oral ragweed consumption. Further activity, presumably toxic effects on the kidney and brain indicating by the significant change of carbamide level and relative organ weights, were also found. Sesquiterpene lactones may also play role in of some beneficial effects of ragweed, however, on the other hand, these compounds may also have cytotoxic effects. The cytotoxic effects of sesquiterpene lactones from ragweed have not been studied on normal human cells. Consumption of ragweed as a medicinal plant or food is questioned by the issues raised as a result of this study. Since there are no human toxicological studies with this plant, the results of this animal experiment should be considered as a warning signal.

## Supporting information

S1 File ARRIVE Guidelines checklist. (DOCX)

S2 File Body weights. (XLSX)

S3 File Amount of cookie dough eaten. (XLSX)

S4 File Detected clinical symptoms. (XLSX)

S5 File Blood chemistry data. (XLSX)

S6 File Absolute and relative organ weights. (XLSX)

S7 File Histopathological pictures and description. (XLSX)
