# Supplementary material for: Repeated-dose toxicity of common ragweed on rats
Source: PLoS One. 2017 May 4;12(5):e0176818. doi: 10.1371/journal.pone.0176818 (PMC5417505; doi:10.1371/journal.pone.0176818)

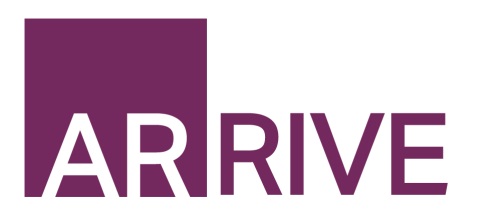


The ARRIVE Guidelines Checklist

Animal Research: Reporting In Vivo Experiments

Carol Kilkenny^1^, William J Browne^2^, Innes C Cuthill^3^, Michael Emerson^4^ and Douglas G Altman^5^

*^1^The National Centre for the Replacement, Refinement and Reduction of Animals in Research, London, UK, ^2^School of Veterinary Science, University of Bristol, Bristol, UK, ^3^School of Biological Sciences, University of Bristol, Bristol, UK, ^4^National Heart and Lung Institute, Imperial College London, UK, ^5^Centre for Statistics in Medicine, University of Oxford, Oxford, UK.*

|  | | ITEM | RECOMMENDATION | Section/ Paragraph |
| --- | --- | --- | --- | --- |
| 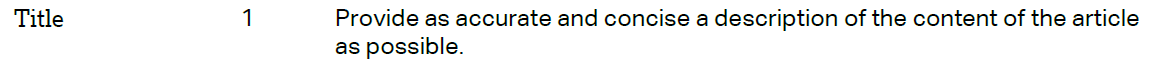 | | | Title |  |
| 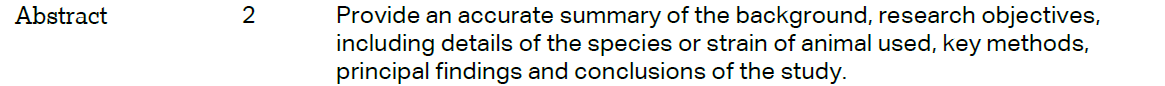 | | | Abstract |  |
| INTRODUCTION | | |  |  |
| 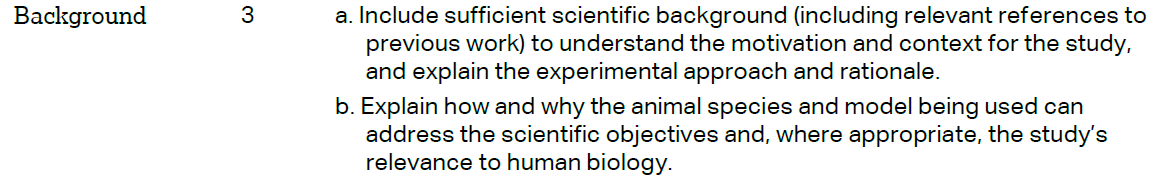 | | | Line 37-73  Line 79-91 |  |
| 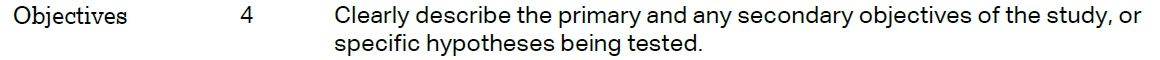 | | | Line 79-91 |  |
| METHODS | | |  |  |
| 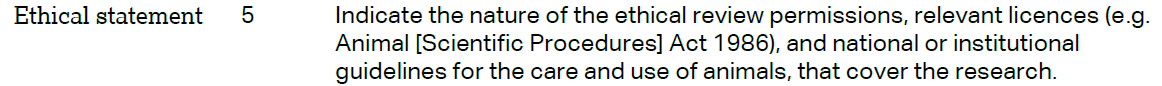 | | | Line 138-143 |  |
| 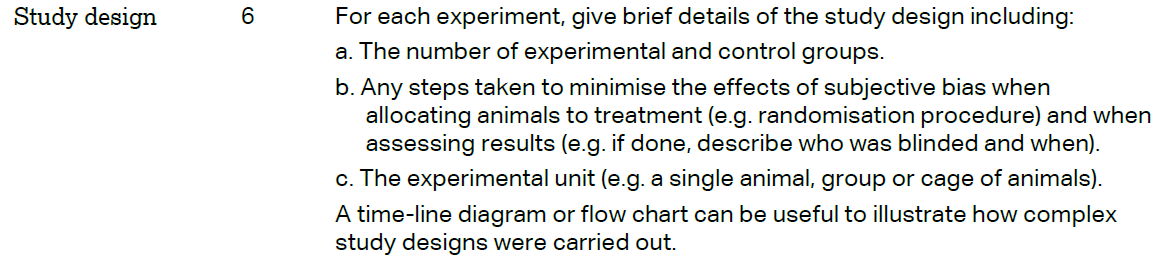 | | | Line 129-132  Line 130-134  Line 129-134  Not relevant |  |
| 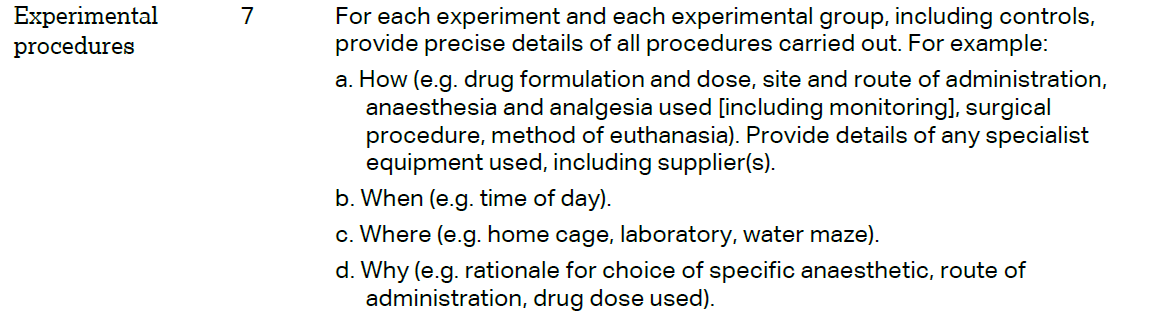 | | | Line 152-167; 174-175; 190-193, S3 Table  Line 148-151  Line 149-151  Line 174-175, 190-193 |  |
| 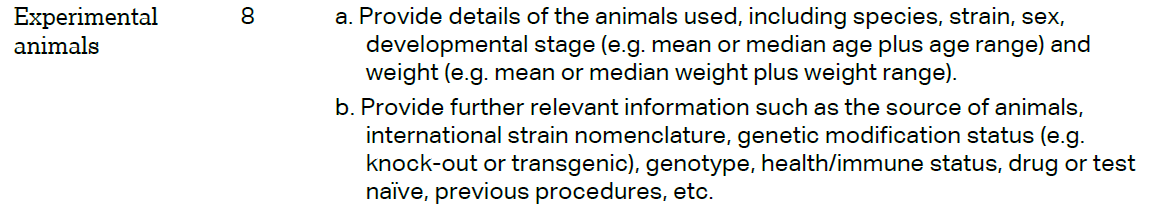 | | | Line 129-130  Not relevant |  |

The ARRIVE guidelines. Originally published in *PLoS Biology*, June 2010^1^

| 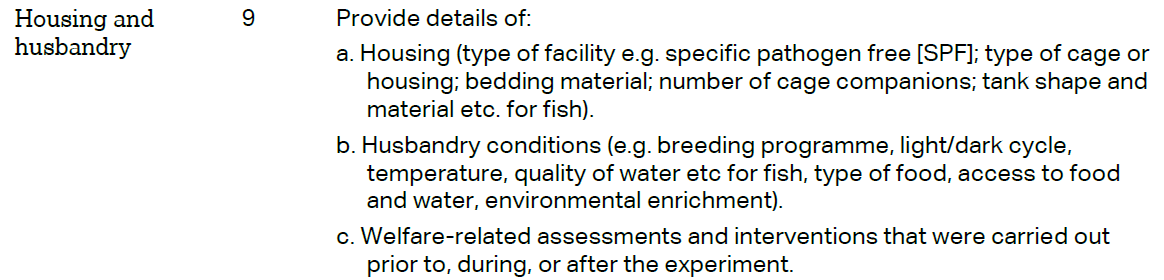 | Line 135-139  Line 135-139  Line 134-135 | |
| --- | --- | --- |
| 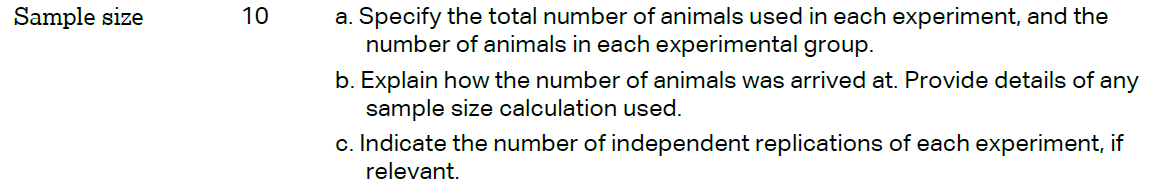 | Line 129, 132  Line 132-134  Not relevant | |
| 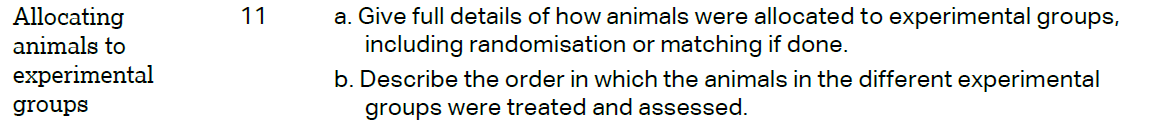 | Line 130-132  Line 149-151, 168-177 | |
| 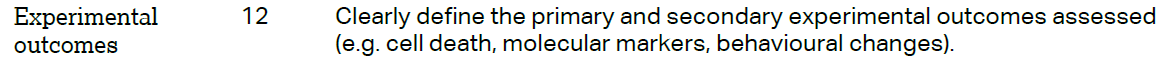 | Line180-181 | |
| 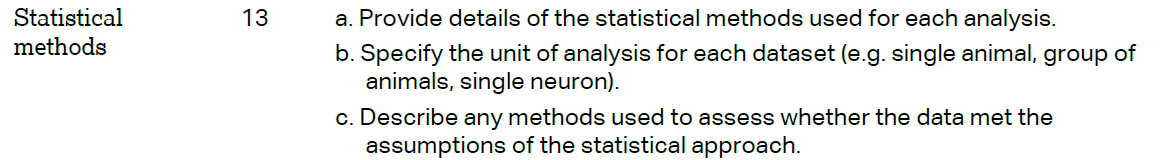 | Line 210-215  Line 214-215  Not relevant | |
| RESULTS |  | |
| 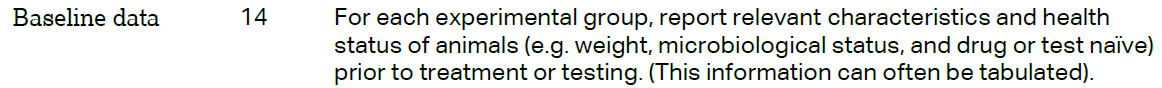 | S2 Table | |
| 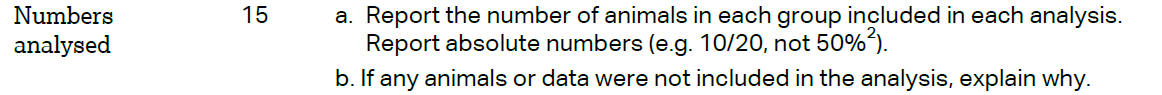 | Line 244, 247, 250, 253, 264, 274  Line 280-286 | |
| 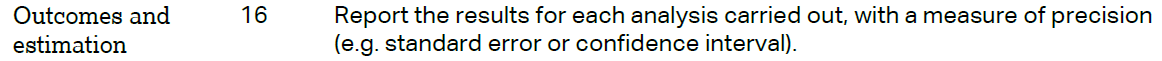 | S2, S3, S5, S6 Tables | |
| 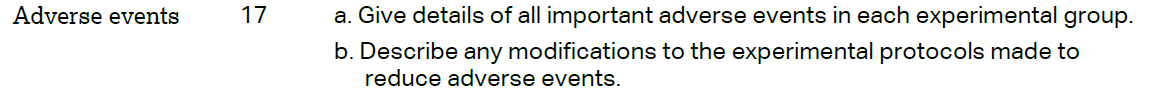 | Line 277-283  Line 283-286 | |
| DISCUSSION |  | |
| 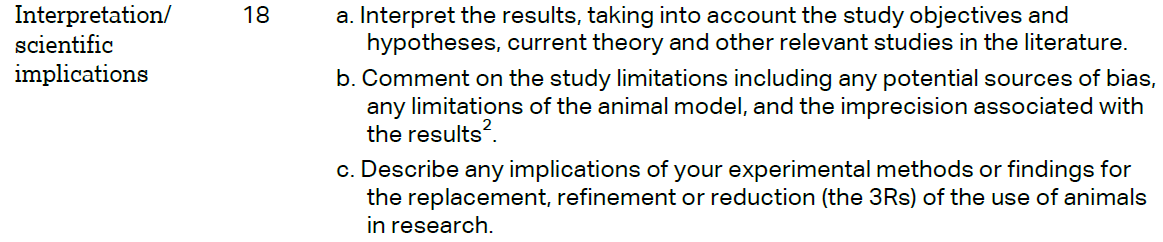 | Line 288-352  Line 353-356  Line 132-134, 174-175 | |
| 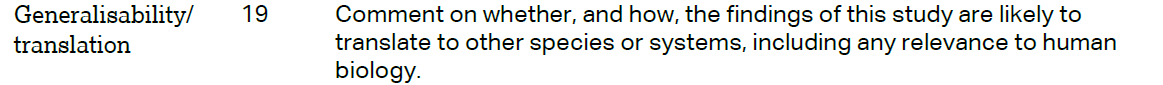 | Line 362-366 | |
| 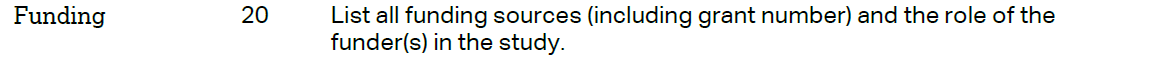 | | Acknowledgement |


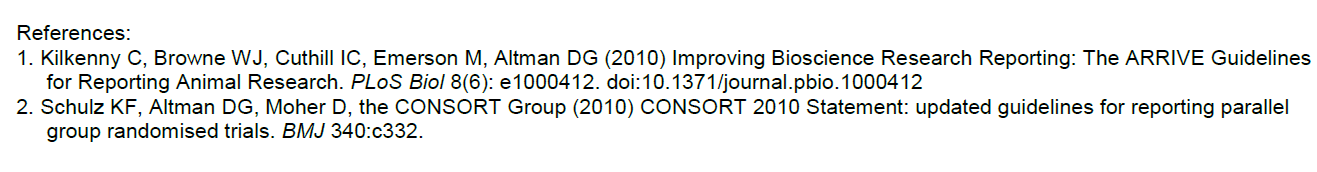

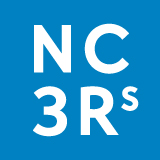

Supplement: S1 File — (DOCX) [file pone.0176818.s001.docx]
